# Testicular microlithiasis in paediatric patients with Klinefelter syndrome from infancy till adolescence: early start of degenerative process in the testes—preliminary results

**DOI:** 10.1007/s00431-022-04663-w

**Published:** 2022-10-25

**Authors:** Dominika Januś, Małgorzata Wójcik, Jerzy B. Starzyk

**Affiliations:** 1 Department of Paediatric and Adolescent Endocrinology, Chair of Paediatrics, Institute of Paediatrics, Jagiellonian University Medical College, Wielicka St. 265, 30-663 Krakow, Poland; 2 Department of Paediatric and Adolescent Endocrinology, University Children’s Hospital, Krakow, Poland

**Keywords:** Testis ultrasound, Testicular microlithiasis, Klinefelter syndrome (KS), Leydig cells, Sertoli cells

## Abstract

To present the results of testicular ultrasonography supported by clinical and hormonal aspects in paediatric patients with Klinefelter syndrome (KS). Prospective analysis of medical files of 20 patients diagnosed with KS between 2016 and 2022. Assessed data included analysis of causes of referral, ultrasound, and clinical characterisation with hormonal evaluation of serum FSH, LH, testosterone, inhibin B, and anti-Müllerian hormone. Non-mosaic Klinefelter syndrome (47, XXY) was diagnosed in 65% of cases (13/20) by the geneticist (including 7 cases prenatally), in 25% (5/20) by the endocrinologist and in 10% (2/20) by the hematologist. Ultrasound assessment revealed bilateral testicular microlithiasis (TM) in all patients. The youngest KS patient with TM was 3 months old. TM patterns have not changed during follow-ups of up to 6 years in any of the patients. In all KS patients markedly reduced echogenicity and in pubertal KS patients, also irregular echostructure of the testes was observed. The hormonal patterns observed in the study group were typical for those already described in KS. Sertoli and Leydig cell function was intact in prepubertal patients and deteriorated after the start of puberty.

*Conclusion*: Although the degenerative process in the testicular tissue starts very early in the testes in KS and is reflected in morphological changes seen in ultrasonography, Sertoli and Leydig cell hormonal function is normal in prepubertal KS patients.

**Table:** 

**What is Known:** *• So far, normal Leydig and Sertoli cell function was observed in infants and prepubertal KS patients.*	
**What is New:** *• The morphological changes in the testes in KS may already be seen in early infancy.*

**What is Known:**

*• So far, normal Leydig and Sertoli cell function was observed in infants and prepubertal KS patients.*

**What is New:**

*• The morphological changes in the testes in KS may already be seen in early infancy.*

## Introduction

Klinefelter syndrome (KS) is a sex chromosome trisomy described in 1942 as ‘a syndrome characterized by gynaecomastia, aspermatogenesis without a-Leydigism and increased excretion of follicle-stimulating hormone’ in adult men by Klinefelter et al. [1]. In 1959, Jacobs and Strong [2] discovered that the presence of an additional X-chromosome is the underlying genetic cause of the syndrome.

The prevalence of KS is 1:660 males [3]. Due to a large variability of phenotypes, diagnosis is often delayed [3, 4]. However, data from 2015 indicate that KS is being diagnosed at an increasingly younger age, with 21% of cases prenatally, 12% during childhood, and 16% during puberty [5].

Infants with KS may show clinical signs of intrauterine hypogonadism such as bilaterally undescended testes and reduced penile length (rarely micropenis) [6]. In prepubertal male patients, the diagnosis is difficult, unless features comprising a wide range of neurocognitive and psychosocial features emerge and whether the patient will be referred for genetic consultation by a neurologist or psychiatrist [6, 7]. Otherwise, symptomless mid- or late-pubertal boys or young adults attract the attention of paediatric endocrinologists and andrologists when specific physical manifestations of the disorder, such as incomplete puberty and fertility issues, arise [3, 8]. The phenotype after the start of puberty typically includes a tall stature, gynoid posture, broad hips, narrow shoulders, gynaecomastia and small testes, development of primary hypogonadism with reduced testosterone in serum, and infertility.

The first description of testicular microlithiasis (TM) was done by Doherty et al. [9] who presented a pattern of small bright echoes in the testes ultrasound with histologic confirmation of TM.

TM is usually incidentally detected by scrotal US as bright, punctuate (usually 1 mm in size), non-shadowing echogenic foci [10, 11]. TM is classified as classic (≥ 5 foci in either or both testes) and limited TM (< 5 echogenic foci in both testes) [12]. TM is thought to be due to abnormal calcification or the presence of debris in the spermatogenic tubules [10]. The etiology of TM within the seminiferous tubules is unknown, although inflammation, defective phagocytosis by Sertoli cells, rapid cell turnover, and an overactive immune response have been suggested as possible mechanisms [13].

The incidence of TM ranges from 0.7 to 12% in children with potential risk factors for primary testicular tumours (PTT) (e.g. testicular pain, testicular masses, personal or family history of TT, undescended testis), and up to 4.2% in asymptomatic children, according to currently published studies [11, 12, 14, 15].

TM may be found in 2.4–5.6% of healthy young men and is as common as 15% among men with an indication for scrotal ultrasound because of scrotal pain, swelling, and a palpable mass, or for a fertility assessment [12, 16–21].

TM is more frequent in adult patients with genetic disorders (e.g. 36.0% in trisomy 21, 17.5% − 33.3% in KS) [22, 23]. There are not many reports on TM in paediatric KS patients [24].

Recently, Fedder [25] questioned the fact that white spots (testicular hyperechogenic foci = THF) that are seen on testicular ultrasounds are in all cases microcalcifications [25].

Ultrasound differential diagnosis of such bright spots includes apart from calcifications also vessel malformations such as hemangiomas, hyperechogenic effect of fat against lymphoid tissue or connective tissue septa, vessels, and nerves [26–28].

The aim of this preliminary prospective one-centre study is to present the results of testicular ultrasonography supported by clinical and hormonal aspects in paediatric patients with KS from infancy till adolescence, as well as to present the probable mechanisms of the structural changes in the KS testes in the light of recent research advances.

## Material and methods

A prospective analysis of medical files and testicular US scans was performed in 20 patients with classic non-mosaic KS (47, XXY) at the mean age of 7.7 years (age range 3 months to 17 years). The analysis included the reason for referral to the doctor, age at KS diagnosis, time of follow-up, routine hormonal status assessing Leydig cell function (LH; testosterone, T), and Sertoli cell function (FSH; AMH; inhibin B) as well as molecular analysis (peripheral blood lymphocytes karyotyping). The karyotypes and routine hormonal evaluation were assessed prior to the inclusion to the study. Karyotypes were established on 40 metaphases from each patient. The volume of the testes was assessed with a Prader orchidometer. Testicular volume (ml) was also calculated using the formula: length × width × height × 0.523 (Table 1).

**Table 1:** Age-dependent clinical and hormonal characterisation of patients. Hormonal evaluation in all patients was ordered by endocrinologists and age of assessment in individual patients is shown in the second and third columns. In adolescents on TRT therapy, hormonal evaluation prior to the start of therapy is presented

***N***	**Age of KS diagnosis (years)**	**Cause/age of referral**	**FSH mIU/ml** **prepuberty *n* < 3.3; puberty *n*: 3–15**	**LH** **mIU/ml** **prepuberty *n* < 3.7; puberty *n*: 1.1–8.3**	**Testosterone** **ng/ml** **prepuberty *n* < 1; puberty *n*: 2.6–10.9**	**AMH** **ng/ml** **prepuberty *n* > 22.9; puberty 0.8–14.5**	**Inhibin B** **pg/ml** ***n*: 25–325**	**Testes volume ml** **Left/right testes texture/penis length** **Tanner stage**	**Testosterone therapy (TRT)**	**Height at first visit** **cm** **[SDS]**
**Mini-puberty**										
1	Prenatally	Referred to endocrinologist 3 months	3.2	2.02	0.96	> 22.9	211	0.36/0.37 Normal Penis normal	-	60 [− 1.2]
2	Prenatally	Referred to endocrinologist age 3.5 months	3.7	1.8	0.78	> 22.9	220	0.38/0.37 Normal Penis normal	-	62 [− 0.9]
3	Prenatally	Referred to endocrinologist age 4 months	2.1	0.9	0.5	> 22.9	172	0.37/0.37 Normal Penis normal	-	64 [− 0.5]
4	Prenatally	Referred to endocrinologist age 4 months	2.5	0.9	< 0.1	> 22.9	184	0.37/0.38 Normal Penis normal	-	59.7 [− 1.3]
5	Prenatally	Referred to endocrinologist age 5 months	3.2	1.1	0.9	> 22.9	178	0.44/0.44 Normal Penis normal	-	66 [− 0.5]
6	Prenatally	Referred to endocrinologist age 5 months	1.9	0.8	< 0.1	> 22.9	194.7	0.45/0.46 Normal Penis normal		64 [− 1.5]
**Prepuberty**										
1	Prenatally	Referred to endocrinologist age 2 2/12 years	1.3	< 0.1	0.1	> 22.9	94	0.31/0.31 Normal Penis normal	-	90 [0.0]
2	1.1	Congenital cardiac defect	< 0.3	< 0.1	< 0.1	> 22.9	135.3	0.3/0.3 Normal Penis normal	-	77 [− 0.01]
3	1.8	Syndactyly of 2nd & 3rd toes	< 0.3	< 0.1	< 0.1	> 22.9	145.2	0.32/0.32 Normal Penis normal	-	84.5 [− 1.0]
4	7	Bone marrow biopsy with karyotyping due to neutropenia age 7 years, tall stature	0.5	< 0.1	< 0.1	> 22.9	161	0.31/0.31 Normal Penis normal	-	135 [+ 2.4]
5	9	Delayed development, motor aphasia	2.6	0.5	< 0.1	> 22.9	119.7	0.33/0.31 Normal Penis normal	-	146.2 [+ 0.5]
6	9	Epilepsy PMH: undescended testes	0.7	< 0.1	0.2	> 22.9	36.8	0.32/.0.33 Normal Penis normal	-	149.2 [+ 0.5]
**Puberty**										
1	13	Tall stature, gynoid posture, broad hips, narrow shoulders	54	16.1	1.2	4	< 4	1.15/1.1 Soft Penis normal GII PIII	-	180 [+ 2.9]
2	13.7	Gynecomastia, gynoid posture, broad hips, narrow shoulders	101.1	38.2	1.4	0.5	< 4	0.64/0.63 Firm Penis normal GII PIII	TRT since 14.3 years	181.7 [+ 0.5]
3	14	Tall stature, gynoid posture, broad hips, narrow shoulders	42.2	17.6	3.9	5	< 4	2.31/2.3 Soft Penis normal GII PIV	-	187.9 [+ 2.7]
4	14.3	Tall stature, gynoid posture, broad hips, narrow shoulders, PMH: undescended testes	29.4	13.3	1.8	0.7	4.3	2.3/1.5 Soft Penis normal GII PIV	TRT since 15 years	198.4 [+ 3.9]
5	16.6	ADHD, atypic autism, gynoid posture, broad hips, narrow shoulders	60.7	30.5	0.7	1.9	** < **4	2.3/2.4 Soft Penis normal GII PIV	TRT since 16.6 years	188.5 [+ 1.6]
6	16.7	Routine pituitary check-up after severe head trauma [15 9/12 years]. Incidental finding of primary hypogonadism, gynoid posture, broad hips, narrow shoulders	20.7	13.4	0.9	2.5	** <** 4	1.5/1.5 Soft Penis normal GII PIV	TRT since 16.8 years	174.6 [− 0.7]
7	16.7	Epilepsy, delayed development, bilateral hypoacusis, gynoid posture, broad hips, narrow shoulders	10.4	8.5	1.1	> 22.9	89.7	2.4/2.3 Firm Penis normal GII PII	TRT since 16.3 years (osteoporosis *Z*-score: − 2.4 SD, bone fracture)	177.6 [− 0.2]
8	17	Ph + chronic myeloid leukemia, bone marrow conventional karyotyping, gynoid posture, broad hips, narrow shoulders	38.7	18.6	1.8	0.2	** <** 4	2.0/2.1 Firm Penis normal GII PIV	TRT since 17 years	189.7 [+ 1.8]

*PMH* past medical history, *G* genitals, *P* pubic hair/pubarche

The pubertal status was assessed by the Tanner scale (G-genitals, P-pubic hair).

**Fig. 1:**
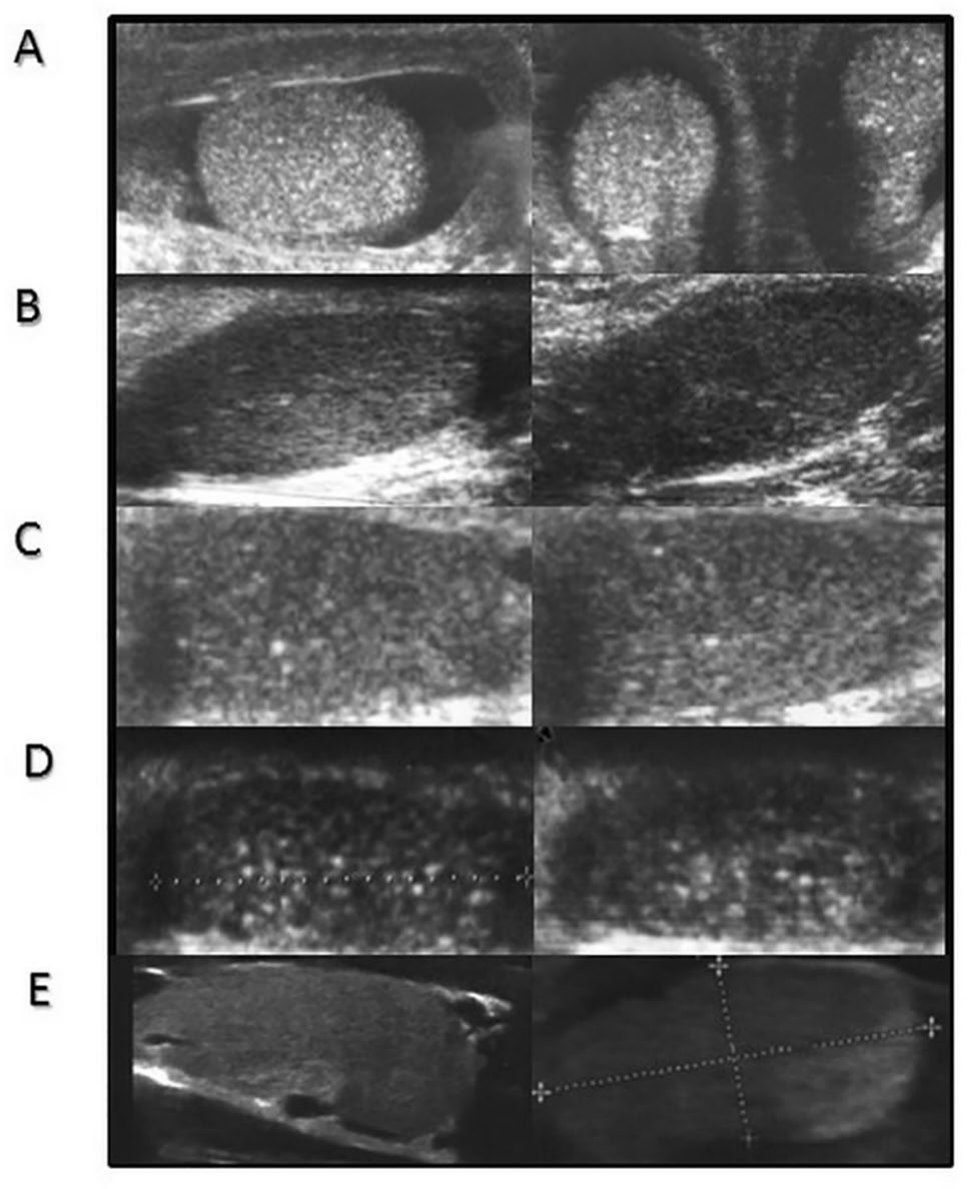
Ultrasound features of testes with TM in the infants from the mini-puberty group. **A** 3 months old, **B** 4 months old, **C** and **D** 5 months old, **E** healthy control 5 months old

**Fig. 2:**
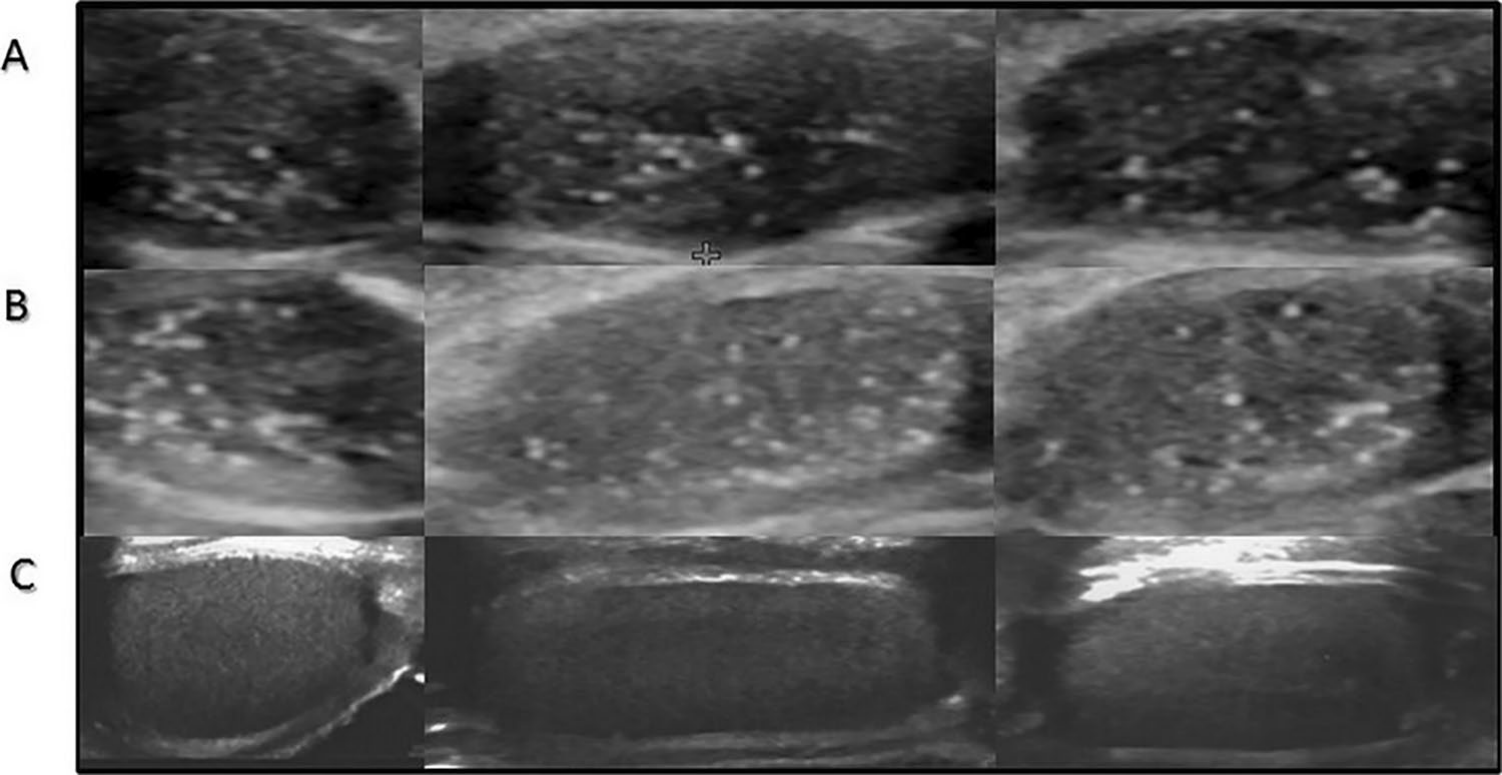
Ultrasound features of testes with TM in patients from prepuberty group. **A** 7.5 years old, **B** 1.8 years old, **C** healthy control 7 years old

**Fig. 3:**
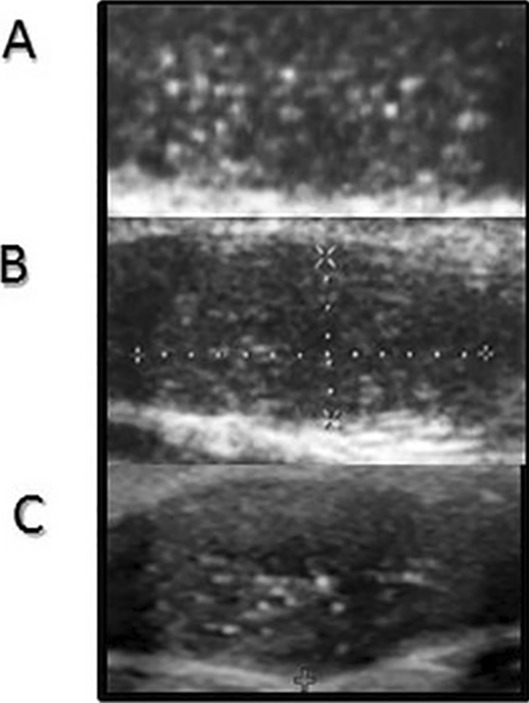
Ultrasound features of the testes during a follow-up of a prepubertal patient. **A** 2017 year (age 2.1 years), **B** 2019 year (age 4.1 years), **C** 2022 year (age 7.1 years)

For analysis of hormonal aspects, the study group was divided into 3 age groups (Table 1; Figs. 1, 2, and 3): group 1: mini-puberty (*n* = 6, age range 3 to 5 months old); group 2: prepuberty (*n* = 6, age range 1.1 to 9 years old); and group 3: puberty (*n* = 8, 13–17 years).

**Fig. 4:**
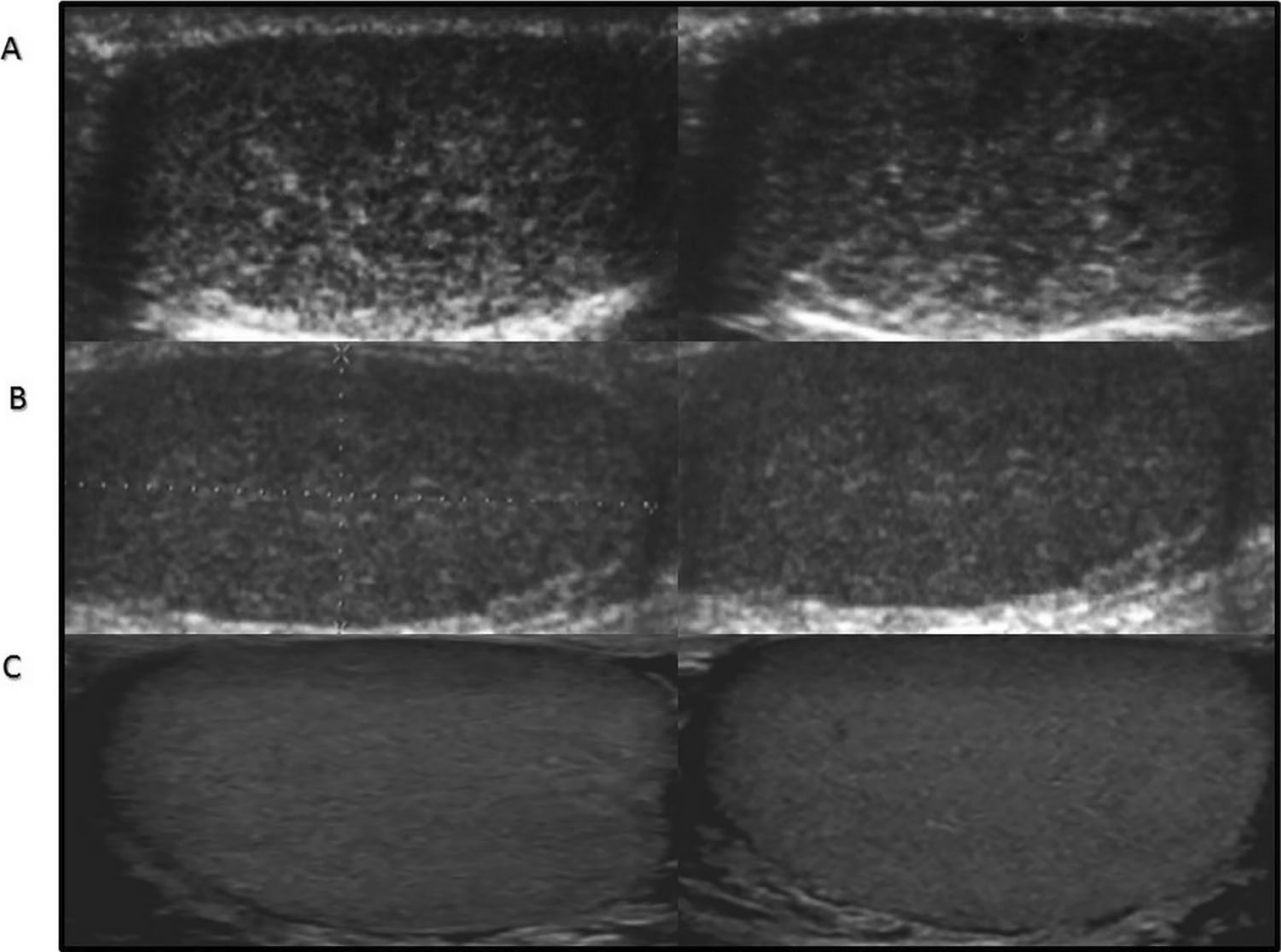
Ultrasound features of testes with TM in pubertal patients. **A** 16.7 years old, **B** 13.7 years old, **C** healthy control 15 years old

Baseline blood samples were obtained from all patients by a venous puncture at 7.00 am after an overnight fast, for determination of serum FSH, LH, testosterone (T), inhibin B (INHB), and anti-Müllerian hormone (AMH).

Plasma T, LH, and FSH were assessed by chemiluminescence immunoassay (ADVIA Centaur XPT Immunoassay System, Siemens, Germany). INHB was assessed by enzymatically amplified two-site two-step sandwich-type immunoassay (ELISA) (Euroimmune 10,535, Oxford Immunotec Global PLC,143 Park Drive, Milton Park, Abingdon, UK). AMH was assessed by an electrochemiluminescence immunoassay analyzer (ECLIA) (Elecsys AMH Plus, Cobas, Roche, Switzerland). The limit of AMH detection with this methodology is 22.9 ng/mL.

All tests were routinely performed in duplicate in the laboratory of the University Children`s Hospital in Krakow, Poland, apart from AMH assessed in a commercial laboratory following the manufacturer’s instructions.

The intra- and interassay coefficients of variation were 3.3 and 4.5% for LH, 3.3 and 5.0% for FSH, 2.0 and 3.1% for T, 3.4% and 6.8% for INHB, and < 9% for AMH. The normal ranges are presented in Table 1.

Ultrasound (US) examination of the scrotum was performed at the University Children`s Hospital in Krakow (Poland) by a board ultrasound-certified paediatric endocrinologist with 23 years of experience in paediatric ultrasonography (DJ). Testicular US was performed using a high-resolution Voluson 730 GE Medical System (8- to 12-MHz linear-array transducer) and Samsung HS40 (LA3-16AD transducer). The US examination was performed in the axial and longitudinal planes in 20 KS patients and in 20 age-matched healthy controls (referred for the assessment of short stature and found to be healthy).

The study was approved by the University Bioethical Commission (positive opinion number: 1072.6120.113.2022). Written informed consent was obtained from the participants and /or their parents.

## Results

Clinical and hormonal data of patients with KS are presented in Table 1.

Non-mosaic KS (47, XXY) was diagnosed in 65% of cases (13/20) by the geneticist, in 25% (5/20) by the endocrinologist, and in 10% (2/20) by the hematologist. Seven out of 20 cases (35%) were diagnosed prenatally by a geneticist. The median height in the mini-puberty group was (-) 1.05 SDS, in prepuberty (-) 0.6 SDS, and during puberty ( +) 1.7 SDS (Table 1).

### Hormonal evaluation

The cross-sectional evaluation of age-related hormonal profiles in the KS study group presents a typical hormonal pattern with deterioration of Sertoli and Leydig cell function at GII PIII pubertal stage and is presented in Table 1. During mini-puberty, a rise of FSH, LH, and testosterone was observed and was within the expected ranges for this period. AMH and inhibin B were increased showing the intact function of Sertoli cells. Testosterone within the age range confirmed normal functioning of Leydig cells. After the period of mini-puberty, the lowering of FSH, LH, and T was observed typically for a prepubertal period with maintained levels of AMH and of inhibin B. In pubertal patients at GII PIII pubertal stage, increased levels of FSH and LH together with decreased AMH and inhibin B concentrations were observed. Six teenagers aged 13.7–17 years presented with overt primary hypogonadism and hormonal signs of Sertoli and Leydig cells insufficiency already at presentation. However, in one of the pubertal patients at GIIPII aged 16.7 years (Table 1), normal function of Sertoli cells was observed, which might be explained by an influence of constitutional delay of puberty observed in this patient.

### Evaluation of testes

In infants and prepubertal boys, the length of the penis and volumes of testes were appropriate for age. In pubertal boys, the maximum volume of the testis was 6 ml by the Prader orchidometer (the volume of one testis). Two patients had orchidopexy due to undescended testes in infancy (Table 1).

### Ultrasound observation

Testicular ultrasound scans from three groups of patients and representative scans from the controls are presented in Figs. 1–3.

TM was found in all patients but not in the controls. The youngest KS patients with TM were 3 to 5 months old (Table 1, Fig. 1). TM was also found both in prepubertal patients above 1 year of age (above the age of mini-puberty) as well as in pubertal (Figs. 2 and 3). In both prepubertal and pubertal KS patients, markedly reduced echogenicity and irregular structure of testes were visible (Figs. 1–3). Irregular echostructure with hypoechogenic areas probably related to the overgrowth of Leydig cells was observed in pubertal KS patients (Fig. 3). The blood flow assessed by Color Doppler and Power Doppler was normal.

The length of follow-up was different in each age group. Patients diagnosed at the age of 16–17 years were followed up till the age of 18 years and then transferred to Adult Andrology Clinic. We have the longest follow-up of 6 years in prepubertal patients. During this follow-up, scrotal ultrasound was performed once a year and we have not seen any changes in the TM pattern (Fig. 4).

### Therapeutic aspects

Therapy with intramuscular prolonged testosterone (testosterone enanthate, T) was started in 6 patients at the mean age of 16 years after overt and symptomatic primary hypogonadism (poor virilisation, low libido, low muscle mass, muscle weakness, gynoid body shape) was confirmed. The testosterone concentrations prior to testosterone therapy (TRT) are presented in Table 1. This therapy was well tolerated by patients and improved their quality of life, school performance, and physical endurance.

## Discussion

In the present study, contrary to other published research articles, we have found TM in testicular ultrasound in all KS patients during mini-puberty, prepuberty, and puberty [24, 29]. The study group was small, but KS is rarely detected in childhood [3, 5]. Although we see more cases detected prenatally during the last years, the overall number of these patients, in one large endocrine centre, has not increased significantly. The complex patient evaluation in our centre includes ultrasonography—point of care, performed by an experienced endocrine paediatric ultrasonographist, enabling early morphological assessments of endocrine glands involving also testicular ultrasonography. In the present study, we were able to report for the first time that although normal Leydig and Sertoli cell function was observed in infants and prepubertal KS patients similarly to other studies, morphological changes in the testes may already be seen in early infancy in some patients [24]. We are aware that a small number of study patients could affect our results but TM was already described in ~ 30% of adult KS patients by Accardo et al. [23].

The discrepancy in results between the studies may also be explained by the fact that the testicular histology is patient-dependent since tubular degeneration and fibrosis can be severe in some patients, while in others, normal tubules can still be observed [30]. Why TM is seen in some but not in all non-mosaic KS patients presumably could be explained by testicular mosaicism [24, 31].

So far, we accepted that in KS patients’ abrupt deterioration of Sertoli cell function takes place during puberty. The finding of TM in infants is of special interest as it confirms that the degenerative process in the testicular tissue starts very early in the testes in KS, probably even prenatally, and continues, as we did not observe any resolution of TM during the follow-up in our group, in the prepubertal period, but cellular functionality was intact for several years until puberty.

Additionally, we have shown decreased echogenicity in testes in all patients and irregular echostructure of testicular tissue in pubertal patients. One explanation may be the rapid progression of tubular and interstitial deterioration in this selected population presenting severe phenotypes.

Morphological abnormalities in the testes in KS patients, the presence of TM and hypoechogenicity, might also be explained by the last research outcomes presenting changes in vascularity found already in prepubertal patients as well as the latest input from molecular studies confirming already prenatal loss of germ cells [32, 33]. Previous studies reported irregular testicular echostructure with hypoechogenic areas thought to be overgrown Leydig cells [29]. In our study, such irregular hypoechogenic areas were seen in pubertal GIIPIV patients with low testosterone concentration in serum. The mechanism of these structural abnormalities might be explained by the following observations. Patients with KS are known to have decreased levels of testosterone; nevertheless, an increased intratesticular testosterone concentration has been found in adult KS patients [33]. The testis transcriptome analysis also revealed increased steroidogenic function of the Leydig cells [34]. This contradiction has been explained by a disturbed vascularity around the Leydig cells in KS testes since reduced artery diameters were previously found in KS men as well as an increased vessel density of the smallest blood vessels (0–100 µm^2^) in prepubertal KS tissue [33, 35, 36]. In a recent in vivo study by Carlomagno et al. [37], contrast-enhanced ultrasonography (CEUS) revealed slower testicular perfusion kinetics that was associated with lower total testosterone levels in subjects with KS [37].

In our study in infants and prepubertal patients with KS, the volumes of testes were appropriate for age. In pubertal patients, the maximum volumes of testes were 6 ml (per testis) and the consistency of testes was mostly soft. As presented in earlier research studies, initially, the testes of KS adolescents grow up to a volume of 6 ml due to the proliferation of Sertoli cells and interstitial cells [38]. The vast majority of 47, XXY spermatogonia cannot complete the meiotic process and become apoptotic [39]. Whether this is specific to the germ cells themselves, or whether it is a malfunction and failure of the Sertoli cells in their nutritive and supporting role or a combination of both, is not fully understood [39]. Each tubule is constricted and either left with few remaining germ and Sertoli cells or devoid of all cells and this degeneration of the interstitial stroma leads to the decrease in the testis volume to a prepubertal size of 2–4 ml [38–41].

The proliferation of germ cells occurs during fetal development and continues into early postnatal life. During this period, primordial germ cells differentiate towards pre-spermatogonia and spermatogonia. Winge et al. [32, 42] proved that this process of differentiation is incorrect in KS fetuses, indicating that germ cell loss already occurs prenatally. Since the loss of spermatogonia begins before puberty, even as early as during fetal development, other mechanisms must be involved in testicular hyalinization and fibrosis that start later, after the start of puberty [43]. Winge et al. [43] by finding markers of the immaturity of somatic cells confirmed previous observations of the role of immature Sertoli and Leydig cells in the adult KS testis. It can be hypothesized that the presence of TM in infants supports the recent molecular research pointing to already prenatal malfunctioning of spermatogenesis in testes. And later on, immaturity of Sertoli and Leydig cells, together with altered vascularity and impaired phagocytosis of cellular debris, leads to the progressive deterioration of testicular function and after a short activation, abrupt primary hypogonadism, hyalinization of seminiferous tubules, and finally infertility [32, 43]. However, hormonal production by Leydig and Sertoli cells is preserved until the beginning of puberty [24].

Histological images of an adult KS testis show significant architectural changes such as fibrosis, Leydig cell hyperplasia, high numbers of degenerated and hyalinized tubules, and absence of germ cells [44]. The question arises: could there be a role of TM in the fibrotic process in KS testes?

It is known that the fibrotic process is initiated after the germ cell loss. However, the exact mechanism leading to testicular fibrosis in KS testes remains unknown [30].

Whether the altered vascularisation also has influence on the occurrence of fibrosis in the KS testes is currently investigated. Recent research is focused on a role of mast cells (MCs) that has been reported in increased numbers in biopsies from infertile patients [45, 46].

It was found that tryptase produced by MCs may be directly involved in the development of the fibrotic thickening of the walls of the seminiferous tubules in infertility, because of the potential of tryptase to induce fibrotic processes [45]. It would appear that altered seminiferous tubules and/or interstitial cells release growth and/or chemotactic factors for MCs [45]. Could TM (a kind of a foreign body) also trigger the release of factors attracting MCs? The mechanisms responsible for the architectural changes of the KS testes and the association with TM require further investigation.

There is not much evidence supporting that TM alone is premalignant; however, TM was strongly associated with increased diagnosis of PTT in children with potential risk factors for PTT [10]. The ultrasound follow-up did not reveal any changes in TM with time nor malignant transformation during the study observation, so we are convinced that TM in KS paediatric patients is benign and can be safely monitored. Even though a high prevalence of TM is found in adults with KS, these men usually have only a few such lesions and the incidence of testicular cancer in KS men is not higher than in other men [47]. Most of the current guidelines note that TM in the absence of solid mass and risk factors for developing PTT does not confer an increased risk of malignant neoplasm and that it does not require further evaluation [48].

Our prospective preliminary clinical study has several limitations. This was an analysis of paediatric patients referred to the tertiary paediatric centre for the differential diagnosis of diversity of symptoms and finally diagnosed with KS, not representing a general paediatric population. Our study group was too small to determine prevalences; however, it differs from others in the fact that TM was found in all KS patients with classic 47, XXY karyotype including small infants.

## Summary

1. The degenerative process in the testicular tissue may start very early in the testes in KS patients.
2. Scrotal US follow-up might be valuable in KS offering early detection of testicular damage and together with clinical and hormonal assessment might be potentially useful in the diagnostic and, maybe in the future, also in the therapeutic process.
